# Immunoglobulin free light chains are biomarkers of poor prognosis in basal-like breast cancer and are potential targets in tumor-associated inflammation

**DOI:** 10.18632/oncotarget.1868

**Published:** 2014-03-26

**Authors:** Tom Groot Kormelink, Desmond G. Powe, Sylvia A. Kuijpers, Abulikemu Abudukelimu, Marcel H.A.M. Fens, Ebel H.E. Pieters, Willemiek W. Kassing-van der Ven, Hany O. Habashy, Ian O. Ellis, Bart R. Blokhuis, Marco Thio, Wim E. Hennink, Gert Storm, Frank A. Redegeld, Raymond M. Schiffelers

**Affiliations:** ^1^ Division of Pharmacology, Utrecht Institute for Pharmaceutical Sciences, Faculty of Science, Utrecht University, Utrecht, The Netherlands; ^2^ Department of Cellular Pathology, Queen's Medical Centre, Nottingham University Hospitals Trust, Nottingham, UK & The John van Geest Cancer Research Centre, School of Science and Technology, Nottingham Trent University, Nottingham, UK; ^3^ Division of Pharmaceutics, Utrecht Institute for Pharmaceutical Sciences, Faculty of Science, Utrecht University, Utrecht, The Netherlands; ^4^ Department of Pathology, Faculty of Medicine, Mansoura University, Mansoura, Egypt; ^5^ Department of Pathology, School of Molecular Medical Sciences, University of Nottingham and Nottingham University Hospitals NHS Trust, Nottingham, UK; ^6^ Laboratory of Clinical Chemistry & Hematology, University Medical Center Utrecht, Utrecht, The Netherlands; ^7^ These authors share equal senior authorship

**Keywords:** immunoglobulin free light chain, mast cell, biomarker, inflammation, tumor progression

## Abstract

Inflammation is an important component of various cancers and its inflammatory cells and mediators have been shown to have prognostic potential. Tumor-infiltrating mast cells can promote tumor growth and angiogenesis, but the mechanism of mast cell activation is unclear. In earlier studies, we demonstrated that immunoglobulin free light chains (FLC) can trigger mast cells in an antigen-specific manner. Increased expression of FLC was observed within stroma of various human cancers including those of breast, colon, lung, pancreas, kidney and skin, and FLC expression co-localized with areas of mast cell infiltration. In a large cohort of breast cancer patients, FLC expression was shown associated with basal-like cancers with an aggressive phenotype. Moreover, lambda FLC was found expressed in areas of inflammatory infiltration and its expression was significantly associated with poor clinical outcome. Functional importance of FLCs was shown in a murine B16F10 melanoma model, where inhibition of FLC-mediated mast cell activation strongly reduced tumor growth. Collectively, this study identifies FLCs as a ligand in the pro-tumorigenic activation of mast cells. Blocking this pathway may open new avenues for the inhibition of tumor growth, while immunohistochemical staining of FLC may be helpful in the diagnosis and prognosis of cancer.

## INTRODUCTION

Inflammation is considered a critical component of tumor progression in some cancer types [1]. The crosstalk between inflammatory cells and tumor cells provides a tumor microenvironment that favors proliferation, invasion, migration and metastasis. The mast cell is an important organizer of the tumor associated microenvironment and inflammatory reactions contained within it [2, 3]. Through the release of cytokines and proteases, mast cells promote angiogenesis and tissue degradation and furthermore, they function as important intermediates in regulatory T cell-induced tolerance [4, 5]. Mast cells are seen infiltrating the periphery of many human tumors such as breast carcinoma, malignant melanoma, colorectal adenocarcinoma, and oral squamous cell carcinoma. Some studies suggest that mast cells may inhibit tumor growth and improve prognosis, evidenced by a lack of correlation between mast cells with angiogenesis and clinical outcome [2, 3, 6-9]. However, other studies indicate that the number of mast cells correlates with an increase in intra-tumoral microvessel density, enhanced tumor growth and invasion, and poor clinical outcome [6, 10-12]. In addition, mast cells can recruit other inflammatory cells including macrophages that are implicated in promoting cancer invasion and metastasis [13, 14]. These findings are supported by recent studies in various experimental tumor models that demonstrate an essential role for mast cells in tumor expansion [10, 15-17].

Mast cells are activated by the crosslinking of IgE and IgG antibodies on specific receptors resulting in the release of vasoactive and inflammatory mediators. Activation can also be induced by other signals, such as complement factors, cytokines, inflammatory mediators, toll-like receptor ligands, and by T-cells [18]. Importantly, we have previously shown that immunoglobulin free light chains (FLCs) can activate mast cells in an antigen-specific manner [19, 20], and along with IgE and IgG, FLCs are an important component of the humoral immune response to antigen exposure.

Recent studies have shown that non-clonal elevation of FLC is a significant predictor of worse overall survival in patients without plasma cell disorders. Interestingly, neoplastic conditions were amongst the most frequent causes of mortality in patients with high polyclonal FLC [21, 22].

We hypothesized that FLC may be involved in the activation of tumor-infiltrating mast cells and thereby influence tumor growth and progression. In this study, we investigated the presence and functional role of FLC in tumor-associated inflammation. In addition, we analyzed the expression of FLC in human cancer of the breast, colon, lung, pancreas, kidney and skin, and we assessed the clinical significance in a large cohort of breast cancer patients with long term clinical follow-up [23].

## RESULTS

### Localization of FLC expression in different human tumor types

**Figure 1:**
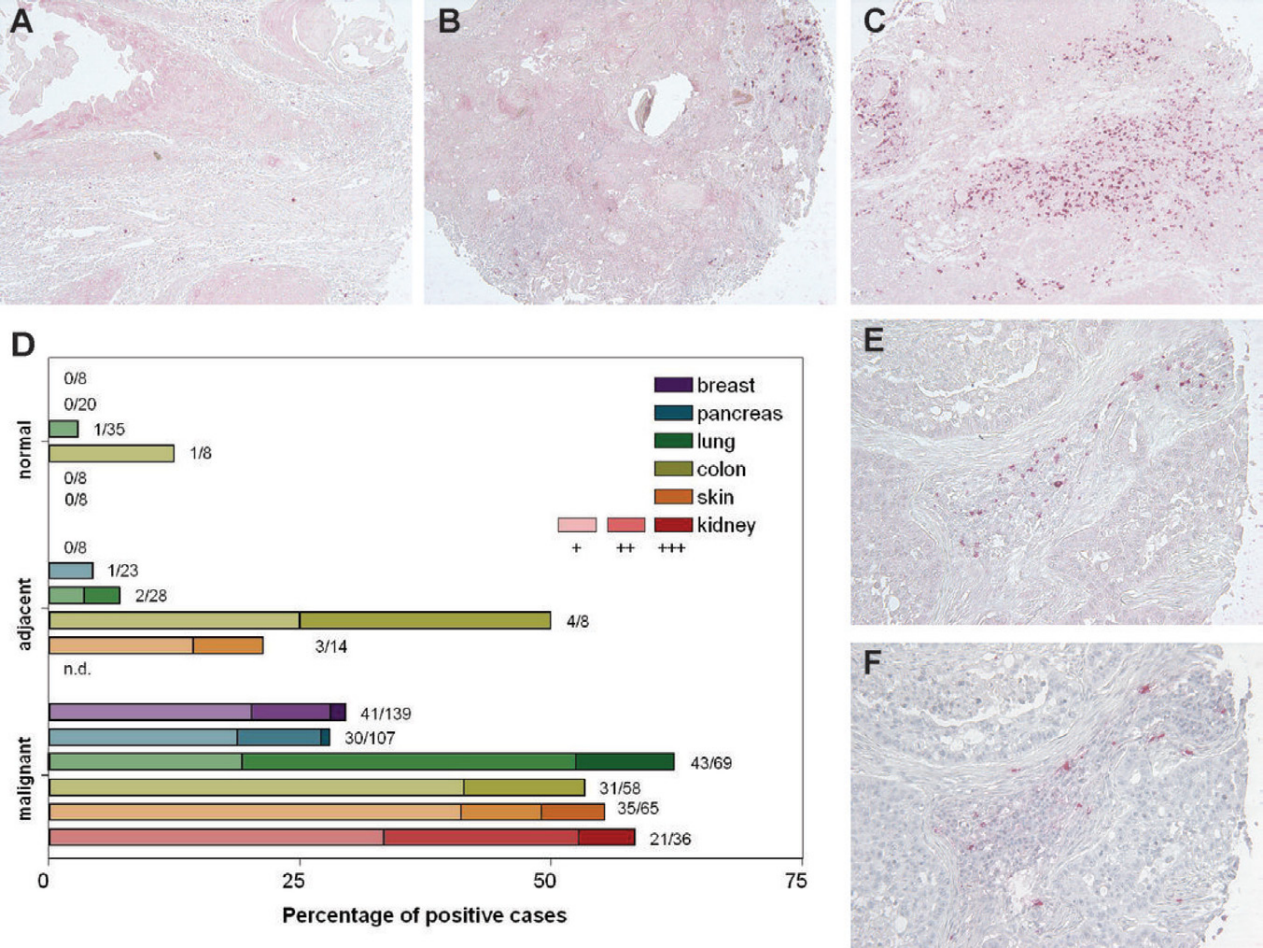
Immunoglobulin free light chains are expressed in breast, pancreas, lung, colon, skin and kidney cancer biopsies (A-C) The FLC staining pattern was discriminated by three categories: +, isolated FLC-positive cells (A); ++, clusters of ≥ 10 FLC-positive cells (B); or +++, clusters of FLC-positive cells encompassing more than 10 % of the tissue core (C) (original magnification, x100). (D) Percentage of FLC-positive malignant, cancer-adjacent or normal tissue biopsies (diameter 1.5 mm) from breast, pancreas, lung, colon, skin and kidney. The numbers next to each bar indicate the number of positive samples divided by the total number of individual patient biopsies examined. The color intensity of the bar corresponds to the degree of FLC staining. Biopsies were evaluated by two researchers who were blinded to the tissue status. (E-F) Expression of FLC (E) and infiltration of mast cells (tryptase staining) (F) were found in same areas in tumor tissue (original magnification, x200).

The cellular localization of kappa and lambda FLC protein expression was investigated in tissue microarrays (TMA) comprising human cancer specimens derived from breast, pancreas, lung, colon, skin, and kidney. Staining was evaluated in 642 samples of tumor tissue, adjacent tissue and normal control tissue (Supplementary Table 1) using a 3-point scoring method: +, isolated FLC-positive cells; ++, clusters of ≥ 10 FLC-positive cells; or +++, clusters of FLC-positive cells encompassing more than 10% of the tissue core (Figure 1A-C). FLC staining was noted in 201 out of 474 cancer tissue cores (42%) (Figure 1D and Supplementary Table 1). Approximately 30% of the breast and pancreatic cancer samples showed FLC positive cells, whereas > 50% of lung, kidney, skin and colon cancer samples were FLC-positive. FLC staining was virtually absent in healthy tissue control samples, only 2/87 control samples showed any positivity. Immunohistochemistry was used to assess the distribution of mast cells and they were found in essentially all tumor samples (143 out of 150 cases (95%)). Frequently, FLCs (Figure 1E) and mast cells (Figure 1F) were present in the same regions supporting co-localization of FLC to this cell type.

### Association of FLC expression and breast cancer

**Figure 2:**
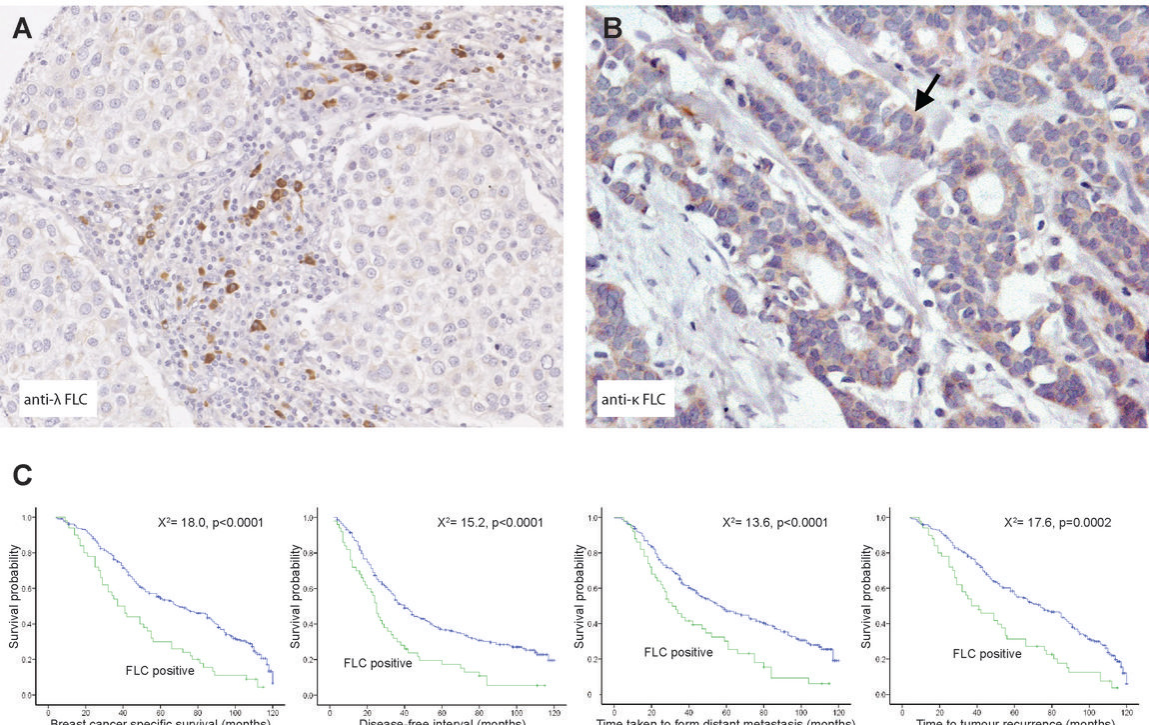
Lambda FLCs are found co-localized to regions of inflammation in breast tumors and their presence predicts worse breast cancer prognosis (A) Using immunohistochemistry Lambda FLC was found localized to inflammatory cells (arrow) located close to medullary breast cancer cells (B) Kappa FLC protein expression was detected in the cytoplasm of ductal breast cancer cells (original magnification, A and B, x100), (C) Kaplan-Meier plots showing patients with lambda FLC positive cells had reduced breast cancer specific survival, reduced time in forming metastases, reduced disease-free interval and reduced time to tumor recurrence.

To investigate the clinical significance of FLC protein expression in human cancer, kappa and lambda FLC expression was analyzed using a breast cancer tissue microarray comprising approximately 700 female patients (aged <75) derived from the Nottingham Tenovus Primary Breast Carcinoma Series (1986-1998) [23]. This well characterized resource contains information on patients' clinical and pathological data and comprises two tissue cores per patient, representing the tumor periphery and centre. A striking differential expression of the FLC isotypes was found. Protein expression of kappa FLC was predominantly localized in the cytoplasm of malignant breast cells and less frequently in inflammatory cells of the adjacent stroma. In contrast, strong lambda FLC expression was seen in stromal inflammatory cells but was only rarely seen in the cytoplasm of cancer cells (Figure 2).

FLC-positive patients showed no significant difference in age or tumor recurrence compared to FLC negative patients. Lambda but not kappa FLC expression was significantly increased in premenopausal patients (p=0.014). Kappa and lambda FLC protein expression showed significant positive correlations with increased tumor size, tumor grade and clinical stage (node involvement), poor NPI+ (Nottingham Prognostic Index), and vascular invasion (kappa p<0.007; lambda p<0.002). FLC positivity was under-represented in the low nuclear grade family of tumor types [30], including tubular cancers, but was increased in ductal and medullary type cancers (p<0.001). Kappa and lambda FLC expression showed significant negative correlations with tumor-relevant markers usually associated with favorable clinical outcome including the hormonal receptor markers for estrogen and progesterone and the apoptotic marker Bcl2 (p<0.001). Instead, significant positive associations were seen with markers of aggressive tumor phenotype such as basal-like markers including cytokeratin (CK) 5/6 and EGFR (p<0.0001), and the proliferation marker MIB1 (p<0.002), mutated p53 and BRCA1 (p<0.0001), and HER2 (kappa: p=0.004).

Multivariate Cox regression (hazards ratio: HR) was performed to test the independence of kappa and lambda FLC protein expression against established prognostic variables including tumor size, stage, grade, vascular invasion, chemotherapy and endocrine therapy, for predicting breast cancer specific survival (BCSS). FLC was not found to be an independent prognosticator of survival. Kaplan-Meier modeling for clinical outcome in the full patient cohort showed no significant associations between kappa FLC protein expression and BCSS (chi-square=2.448, *p*=2.448), disease-free interval (DFI) (chi-square=2.222, *p*=0.136), shortened time for metastases formation (DM) (chi-square=2.234, *p*=0.135), or tumor recurrence (chi-square =1.307, *p*=0.253). However, lambda FLC expression showed a highly significant correlation with decreased BCSS (chi-square=18.0, *p*<0.0001), DFI (chi-square=15.2, *p*<0.0001), DM (chi-square=13.6, *p*=0.0002), and tumor recurrence (chi-square=17.6, *p*<0.0001) (Figure 2C).

### The functional role of FLC in supporting tumor growth in a mouse melanoma model

**Figure 3:**
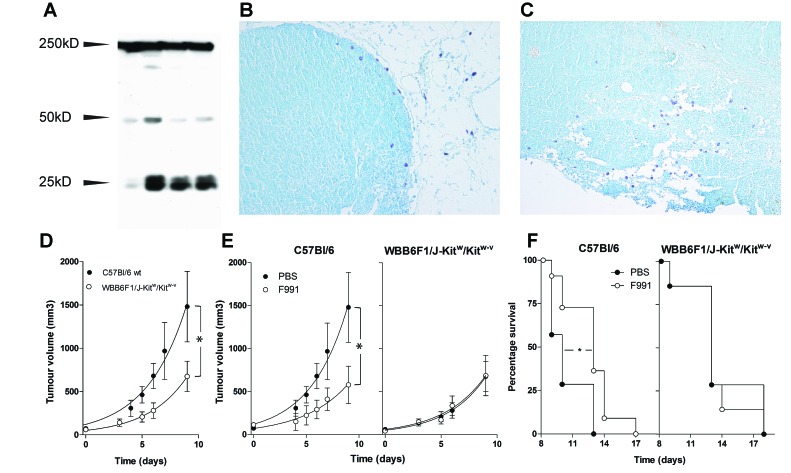
FLCs are responsible for mast cell activation supporting tumor growth of B16F10 melanoma (A) Immunoblot analysis of FLC monomers (25 kD) and dimers (50 kD) in homogenized B16F10 tumor tissue. Each lane represents a tumor isolated from an individual animal. (B-C) Toluidine blue staining of mast cells in B16F10 melanoma tissue showing peri-tumoral and intra-tumoral mast cells. All of the melanomas were isolated from B16F10 inoculated wild type mice. (D-F) Mast cell deficiency and the FLC antagonist F991 attenuate B16F10 melanoma growth. (D) Tumor growth in PBS-treated wild type C57Bl/6 (closed circles) and mast cell-deficient WBB6F1/J-Kit^W^/Kit^W-v^ (open circles) mice. (E) Tumor growth in wild type C57Bl/6 (left) and mast cell-deficient WBB6F1/J-Kit^W^/Kit^W-v^ (right) mice treated weekly with 20 µg F991 (open circles) or PBS (closed circles) intra-tumorally. Data are the mean ± SEM, *n* = 10 animals/experimental group, * indicates *p* < 0.05 by comparing total areas under the curve (D and E). (F) Time-to-reach-humane-endpoint in wild type C57Bl/6 (left) and mast cell-deficient WBB6F1/J-Kit^W^/Kit^W-v^ (right) mice treated weekly with 20 µg F991 (open circles) or PBS (closed circles) intra-tumorally. *n* = 10 animals/experimental group, * indicates *p* < 0.05. Effects of F991 were tested in 4 independent replicate experiments.

In earlier work we have shown that mast cells can be activated by FLCs (14,15). The functional importance of the association between FLCs and mast cells in tumor development and growth was further investigated in an *in vivo* melanoma mouse model, in which tumor-associated inflammation is an important driver for tumor growth [31]. Using western blotting, we demonstrated the presence of FLC proteins in subcutaneously implanted B16F10 melanoma in C57Bl/6J mice (Figure 3A). The tumor tissue contained monomeric (25 kDa) and dimeric (50 kDa) forms of FLC. Isolated B16F10 melanoma cells did not produce FLCs *in vitro* (data not shown). Mast cell infiltration, a prominent feature of B16 melanoma models [9, 32, 33], was also observed, especially at the tumor periphery using toluidine blue staining (Figure 3B-C).

The possible functional role of FLCs and mast cells in tumor growth in the B16F10 melanoma model was investigated both in wild type C57Bl/6J mice that possess normal numbers of mast cells and mast cell-deficient WBB6F1/J-Kit^W^/Kit^W-v^ mice. After subcutaneous melanoma cell inoculation, the average time for the tumors to become palpable was 7.7 ± 0.5 days in C57Bl/6J mice (mean ± SD, *n* = 20) and 7.5 ± 1.4 days for WBB6F1/J-Kit^W^/Kit^W-v^ mice (mean ± SD, *n* = 20). However, subsequent tumor growth was greatly reduced in WBB6F1/J-Kit^W^/Kit^W-v^ mice (*p*=0.03 comparing areas under the tumor growth curve), confirming a critical role for mast cells in B16F10 melanoma tumor growth (Figure 3D) [9, 32, 33]. The role of FLC-induced mast cell activation in supporting tumor growth was further investigated by using a specific peptide FLC antagonist (F991) [19]. When tumors were palpable, the animals were treated intra-tumorally with weekly doses of 20 μg F991 or with vehicle (PBS) alone. In the wild type C57Bl/6J mice, F991 greatly reduced tumor growth when treated with F991. The vehicle-treated wild type animals were culled at day 9 after the first treatment because they had reached the humane endpoint (tumor volume≥1500 mm^3^). F991-treated wild type animals showed an average 61% lower tumor volume (*p*=0.03) and a 49% reduction in growth rate (*p*=0.02; total area under the tumor growth curve) compared to non-F991 treated control animals (Figure 3E). Four replicate experiments were performed and the volumes of F991-treated tumors were between 52 and 76% smaller than those of controls. The median time-to-reach-the-humane-endpoint in F991-treated wild type mice was also increased to 13 days compared to 10 days in vehicle-treated wild type controls (*p*=0.03, log-rank (Mantel-Cox) test) (Figure 3F). As expected, F991 did not affect tumor growth in mast cell-deficient WBB6F1/J-Kit^W^/Kit^W-v^ (Figure 3E), nor did it affect the median survival time in mast cell-deficient animals (Figure 3F).

## DISCUSSION

In this study, we provide evidence for the involvement of FLCs in cancer pathology. FLC protein expression was detected in tumor-associated tissue from human breast, pancreas, lung, colon, skin and kidney, while FLC staining was virtually absent in comparative healthy tissue. The production of FLC was co-localized with mast cell infiltrates in tumor tissue leading to the proposal that FLC can activate mast cells occurring in peri-tumoral inflammation. The clinical significance of FLC isotype expression was studied in a well-defined large cohort of female breast cancer patients with long-term follow-up. We showed that FLC expression is associated with an aggressive tumor trait, particularly those with a triple-negative (estrogen receptor, progesterone receptor and HER2 negative) basal-like phenotype. In addition, FLC expression correlated with several molecular markers of poor disease prognosis evidenced by increased cell proliferation, reduced apoptosis and p53 mutation. Recently, triple-negative and aggressive breast cancer signatures have been associated with specific inflammatory mediators [34, 35]. Strikingly, whereas kappa and lambda FLC both indicated aggressive tumor features, only lambda FLC showed a significant reduction in breast cancer-specific survival, disease free interval, time to metastasis, and time for tumor recurrence. This suggests that these FLC positive cells, possibly mast cells because of its co-localization with regions of mast cell infiltration, are markers of high risk patients needing more intensive clinical management. The biological significance of kappa FLC localization in neoplastic epithelium is not apparent and requires further study. Recently, it was shown that IgG can be produced by breast cancer cells [36], which could possibly attribute to the presence of kappa FLC in malignant breast cancer cells observed here. Our study indicates that micro-localization of the expression of FLC in tumor tissue near peri-tumoral inflammatory cells is of importance for aggravated tumor pathology and poor clinical outcome. To our knowledge this is the first study to show the different localized expression of kappa and lambda FLC isoforms in tumor tissue. Further studies are needed to understand its relation to inflammation-driven tumor development and growth.

A possible functional importance of FLC to tumor growth was further explored in a murine model of human malignant melanoma using B16F10 cells. This tumor model responds to anti-inflammatory therapy [31] and is dependent on mast cells [9, 32, 33]. In this study, it was shown that FLC expression occurs associated with these murine tumors as well. Inhibition of FLCs by the antagonist F991, a peptide which binds to FLC [19, 37], greatly reduced melanoma tumor growth. F991 specifically prevents mast cell activation by FLCs, but has no effect on activation by IgG or IgE [19] nor does F991 directly activate mast cells to induce degranulation (data not shown). Current data suggest that FLC may activate tumor-associated mast cells to produce mediators that enhance tumor growth, or that activated mast cells instruct the production of autocrine growth factors by the tumor. Furthermore, the reduced tumor growth in mast cell deficient-mice and the unresponsiveness of B16F10 melanoma in these mice to F991 treatment further underscores a critical role of FLC-mediated mast cell activation on inflammation in tumor progression. These observations suggest that using a FLC receptor antagonist such as F991 could offer a novel therapeutic approach for slowing cancer growth and improving specific survival time.

As B16F10 melanoma cells do not produce FLCs *in vitro*, indicating that systemic FLCs infiltrate into the tumor, or that local B cells or plasma cells are a source of FLCs. B cell infiltration and antibody production have been shown to precede premalignant transformation and to enhance tumor proliferation [27, 38].

A number of different biological mechanisms have been proposed to explain how mast cells affect tumor growth, predominantly in experimental tumor models [2, 3, 6-8, 17]. The effect of mast cell activation at the tumor site appears to depend on tumor localization (intra- or peri-tumoral) [39], stimulating factor [9], and degree of mast cell activation [40, 41]. Timing is also important. Mast cells entering the tumor microenvironment at an early stage appear to promote angiogenesis which is crucial for tumor growth [15, 42-44]. In addition, mast cells can induce an immunosuppressive tumor microenvironment [17] by directly enhancing regulatory T cell (Treg) function via mast cell-T cell contact [44], IL-10 [46], tryptophan metabolism [47], amphiregulin production [48], or adenosine [44], or indirectly by inducing tolerogenic dendritic cells [5]. Furthermore, mast cells serve in attracting other inflammatory cells including macrophages that are implicated in initiating cell signaling events involved in colonic tumor invasion [13] and breast cancer metastasis [14].

In our previous studies, FLC-mediated mast cell activation has been associated with the induction of several inflammatory disorders [19, 49, 50]. Present data suggest that FLC could contribute to tumor growth. It remains to be determined if FLC-induced mast cell activation is mediated via induction of e.g. immune tolerance, stimulation of angiogenesis and/or release of other pro-tumorigenic factors. Because FLC are able to bind antigen with reasonably high affinities [20] and antigen-specific FLC can be detected in human patients (manuscript in preparation), it will be interesting to determine the antigen-specificity of the FLC present in tumor tissues in future research. Alternatively, FLC-binding to mast cells without subsequent antigen binding might result in selective mediator release as was shown for IgE, which enhances VEGF release and thereby contributes to melanoma tumor growth [51].

In conclusion, our study suggests that FLC activated mast cells are associated with increased tumor growth in a preclinical tumor model. Increased FLC expression in stroma of breast cancer tissue showed to be associated with reduced specific survival. This paradigm appears to extend to other tumor types too. Blocking FLC with the antagonist F991 demonstrates a novel strategy for therapeutically modifying mast cell-mediated cancer growth.

## MATERIALS AND METHODS

Detection of FLC-positive cells and mast cells in tissue arrays of different human tumor types tissue array slides from US Biomax (Rockville, MD) were stained using the Envision G2 System/AP (rabbit/mouse; DakoCytomation) according to manufacturer's instructions. Separate sections were stained for kappa and lambda FLCs simultaneously [24] (Fκ-C8 and Fλ-G9 purchased from Dr. A. Solomon, Tennessee; both 1 μg/mL) and mast cell tryptase (clone AA1, DakoCytomation; 0.4 μg/mL). The specific recognition of κ and λ FLCs by mAbs Fκ-C8 and Fλ-G9 in immunohistochemistry has been demonstrated earlier by Davern et al [24].

Primary antibodies were diluted in Tris-buffered saline containing 0.1% Triton X-100 and 1% bovine serum albumin. For tryptase staining, tissue deparaffinization was followed by heat-induced epitope retrieval using citrate buffer (10 mM citric acid containing 0.05 % Tween-20, pH 6). Slides were counterstained with hematoxylin. Sections were viewed using an Eclipse TE2000-U inverted microscope with 4× and 40× objectives (Nikon). Images were analyzed using NIS elements BR 2.3 software (Nikon). FLC staining was differentiated as follows: – (absence of staining), + (isolated cells with positive staining for FLC), ++ (clusters of ≥ 10 cells with positive staining for FLC), or +++ (clusters of cells encompassing > 10 % of the tissue core with positive staining for FLC). Staining was scored by two researchers who had no knowledge of biopsy status.

### Patient studies

#### Patient selection for detailed analysis of FLC expression in breast cancer

Tissue microarray (TMA) slides from the Nottingham Breast Cancer group were used in this study comprising approximately 700 patients from women aged 70 or less derived from the Nottingham Tenovus Primary Breast Carcinoma Series (1986 and 1999). This well characterized resource contains information on patients' clinical and pathological data including histologic tumor type, primary tumor size, lymph node status, histologic grade, and data on other breast cancer relevant biomarkers [23]. Patients within the good prognostic group (Nottingham Prognostic Index (NPI) ≤3.4) did not receive adjuvant therapy (AT) [25]. Hormonal therapy (HT) was prescribed to patients with ER-α+ tumors and NPI scores of >3.4 (moderate and poor prognostic groups). Pre-menopausal patients within the moderate and poor prognostic groups were candidates for CMF (Cyclophosphamide, Methotrexate, and 5-Flourouracil) chemotherapy. Conversely, postmenopausal patients with moderate or poor NPI and ER-α+ were offered HT, while ER-α- patients received CMF if fit. Survival data including survival time, disease-free interval (DFI) and development of loco-regional and distant metastases (DM) were maintained on a prospective basis. Median follow up was 124 months (range 1 to 233). Breast cancer specific survival (BCSS) was defined as the time (in months) from the date of the primary surgical treatment to the time of death from breast cancer. DFI was defined as the interval (in months) from the date of the primary surgical treatment to the first loco-regional or distant metastasis.

This study was approved by the Nottingham Research Ethics Committee 2 under the title “Development of a molecular genetics classification of breast cancer”. Patients consented to tissue samples being used for research purposes.

#### Immunostaining of human breast cancer tissue arrays

The localization and number of kappa and lambda FLC protein expressing cells was assessed in formalin fixed paraffin embedded (FFPE) tissue microarrays of breast cancer to characterize their association with cancer-relevant biological markers and with clinicopathologic features. The cut-offs used for categorizing the various biomarkers have been previously described [26]. HER2 scoring was performed using the Hercept tests guidelines (DakoCytomation, Cambridge, UK).

Immunohistochemistry (IHC) was performed using a DakoCytomation Techmate 500 plus (DakoCytomation, Cambridge, UK) instrument with a linked streptavidin biotin technique and DAB chromogen as previously described [27]. Primary antibodies were optimized on full face FFPE sections and TMAs of breast cancer tissue. Negative controls comprised omission of the primary antibody and substitution with an inappropriate primary antibody of the same Ig class. After dewaxing, sections were subjected to microwave antigen retrieval using 0.01M citrate buffer, pH6 and then immunostained using the optimized 1:50 dilution of mouse monoclonal kappa (Fκ-C8) and separately, lambda FLC (Fλ-G9) Ab [24].

Staining intensity was subjectively assessed (DGP & HOH) for each marker according to a three point scoring system comprising: 0 (negative); 1 (weak); or 2 (strong) staining intensity confined to the malignant tissue or stromal inflammatory cells. Cases were scored without knowledge of patient outcome.

#### Univariate and Multivariate Statistics

The results have been reported according to REMARK criteria that establish a framework for reporting tumor marker prognostic studies [28]. The association between FLC and other tumor-relevant markers was assessed using Chi square test. Association with clinical outcome including BCSS, DFI, DM formation, and local tumor recurrence was modeled using Kaplan-Meier plots (Version 15, SPSS Inc, IL, USA) with the log rank (Mantel-Cox) test. A p-value of less than 0.05 was deemed significant with 95% confidence intervals.

### Animal studies

#### Detection of FLCs and mast cells in mouse B16F10 melanoma tissue

B16F10 melanoma tumors were developed subcutaneously in mice, as described previously [29]. In brief, 10^6^ B16F10 cells cultured *in vitro* were injected subcutaneously into the flank of C57Bl/6J wild type or mast cell-deficient WBB66FI/J-Kit^w^/Kit^W-v^ mice. For mast cell detection, tumors were fixed in 10% buffered formaldehyde and embedded in paraffin. Deparaffinized sections were stained with toluidine blue solution. FLCs were detected by western blotting. For these experiments, mouse tumors were collected and immediately homogenized and lysed with MT Cell lysis reagent containing a protease inhibitor cocktail (Sigma-Aldrich, the Netherlands). The lysed sample was centrifuged for 10 min at 20000 × *g* to pellet the tissue debris, and the protein supernatant was subjected to western blotting (iBlot; Invitrogen, Frederick, MD). Horseradish peroxidase-labeled goat anti-mouse kappa light chain (0.1 μg/mL, SouthernBiotech, Birmingham, USA) was used to immunostain the membranes.

#### Treatment with the peptide antagonist F991

C57Bl/6J wild type or mast cell-deficient WBB66FI/J-Kit^w^/Kit^W-v^ mice that received B16F10 cells via subcutaneous flank injection were monitored for tumor growth. At the time the tumor became palpable, 25 µl PBS containing 20 µg F991 or vehicle alone was injected in the tumor vicinity. Treatment was repeated weekly. Tumor growth was monitored by measuring the largest and smallest superficial diameters of the tumors using digital calipers. The tumor volume was calculated as follows: (0.52 × largest diameter) × (smallest diameter^2^). Animals were considered to have reached the endpoint of the experiment when the tumor volume measured ≥ 1500 mm^3^.

## SUPPLEMENTARY FIGURES AND TABLE


