# Supplementary material for: Immunoglobulin free light chains are biomarkers of poor prognosis in basal-like breast cancer and are potential targets in tumor-associated inflammation
Source: Oncotarget. 2014 Mar 26;5(10):3159–67. doi: 10.18632/oncotarget.1868 (PMC4102799; doi:10.18632/oncotarget.1868)
Supplement: Supplementary file 1 [file oncotarget-05-3159-s001.pdf]

Immunoglobulin free light chains are biomarkers of poor prognosis in basal-like breast cancer and are potential targets in tumor-associated inflammation

Supplementary results

Supplementary Table 1. FLC staining in biopsies of human malignant tissue in various organs compared to staining in tissue adjacent to the tumor and normal tissue.

			number	+++	++	+	-
**Breast**			**number**	**+++**	**++**	**+**	**-**
	**Malignant**		139	28	11	2	98
		Metastatic carcinoma	*26*	*6*	*4*		*16*
		Invasive ductal carcinoma	*65*	*17*	*5*	*2*	*41*
		Lobular carcinoma	*20*	*1*	*1*		*18*
		Intraductal carcinoma	*20*	*4*	*1*		*15*
		Squamous cell carcinoma	*4*				*4*
		Lobular carcinoma in situ	*4*				*4*
	**Adjacent**		8				8
	**Normal**		8				8
**Pancreas**							
	**Malignant**		107	20	9	1	77
		Ductal adenocarcinoma	89	19	9	1	60
		Adenosquamous carcinoma	3	1			2
		Islet cell tumor/carcinoma	11				11
		Metastatic carcinoma	4				4
	**Adjacent**		23	1			22
	**Normal**		20				20
Lung							
	Malignant		*69*	*13*	*23*	*7*	*26*
		Adenocarcinoma	*19*	*6*	*6*	*3*	*4*
		Alveolar carcinoma	*1*		*1*		
		Squamous cell carcinoma	*42*	*7*	*16*	*4*	*15*
		Small cell undifferentiated carcinoma	*7*				*7*
	Adjacent		*28*	*1*	*1*		*26*
	Normal		*35*	*1*			*34*
**Colon**							
	**Malignant**		58	24	7		27
		Adenocarcinoma	*18*	*4*	*4*		*10*
		Mucinous adenocarcinoma	*14*	*6*			*8*
		Carcinoid	*2*	*1*			*1*
		Metastatic carcinoma	*20*	*10*	*3*		*7*

	Papillary adenoma	*4*	*3*			*1*
**Adjacent**		8	2	2		4
**Normal**		8		1		7
**Skin**						
**Malignant**		65	26	5	4	30
	Squamous cell carcinoma	*47*	*17*	*2*	*1*	*27*
	Basal cell carcinoma	*11*	*6*	*2*	*1*	*2*
	Melanoma	*7*	*3*	*1*	*2*	*1*
**Adjacent**		14	2	1		11
**Normal**		8				8
**Kidney**						
**Malignant**		36	12	7	2	15
	Clear cell carcinoma	*11*	*4*	*2*	*1*	*4*
	Granular cell carcinoma	*12*	*4*	*4*		*4*
	Transitional cell carcinoma	*20*	*4*	*1*	*1*	*7*
**Adjacent**		n.d.				n.d.
**Normal**		8				8

n.d. not determined
